# A Needle‐Like MOFzyme with High Aspect Ratio and Extended pH/Temperature Working Ranges for Total Antioxidant Capacity Determination and Oxidant‐/Light‐Free Dye Degradation

**DOI:** 10.1002/open.70196

**Published:** 2026-04-24

**Authors:** Hanieh Borhanipasvisheh, Anahita Barghi, Peyman Abazari

**Affiliations:** ^1^ Department of Inorganic Chemistry Faculty of Chemistry University of Guilan Rasht Iran; ^2^ Institute of Agricultural Life Science Dong‐A University Busan Republic of Korea; ^3^ Department of Analytical Chemistry Faculty of Chemistry University of Kashan Kashan Iran

**Keywords:** biosensing, enzyme‐free biosensors, MOFs with high aspect ratio, needle‐like MOFzyme, total antioxidant capacity

## Abstract

A needle‐like MOFzyme with high aspect ratio and extended pH/temperature working ranges was developed for quantification of total antioxidant capacity (TAC) and oxidant‐/light‐free dye degradation. The MOFzyme showed a length, width, and high aspect ratio of 17 ± 8 µm, 1.5 ± 0.6 µm, and 11.33, respectively. A superb oxidase‐like activity, an enhanced *V*_max_ of 0.14 nM s^−1^, an improved *K*_m_ of 0.12 mM, a wide pH range of 3.0–8.0, and an extended thermal range of 25–70°C were obtained, revealing their practical applicability for developing pH‐ and temperature‐independent protocols. The MOFzyme was then utilized for the determination of TAC using trolox as a model antioxidant, providing a linear range of 0.0–200.0 μM, and a very low detection limit of 1.0 μM was obtained for TAC quantification. The selectivity studies showed that other coexisting materials cannot interfere with sensor response. The intra‐day and inter‐day reproducibility assays showed a %RSD as low as 1.7% and 2.36% for the sensor. The dye degradation was performed using rhodamine B as a model molecule, revealing 99.7% degradation within 40.0 min without any oxidant/light irradiation and 10 cycles of reusability. The applicability of the MOFzyme for real food and sustainable environmental applications was proved.

## Graphical abstract

A needle‐like MOFzyme with high aspect ratio and extended pH/temperature working ranges was developed for quantification of total antioxidant capacity and oxidant‐/light‐free dye degradation.

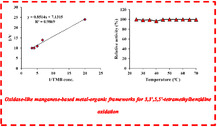

**Abbreviations**
ABTS: 2,2′‐Azino‐bis(3‐ethylbenzothiazoline‐6‐sulfonic acid
DAB: 3,3′‐Diaminobenzidine
EDX: Energy‐dispersive X‐ray
MOF: Metal–organic frameworks
OPD: o‐Phenylenediamine dihydrochloride
SEM: Scanning electron microscopy
TAC: Total antioxidant capacity
TMB: 3,3′,5,5′‐Tetramethylbenzidine

## 1 Introduction

Enzyme catalysis is a highly selective and efficient process that involves the binding of a substrate to a specific region on the enzyme known as the active site [1, 2, 3]. This proximity triggers catalysis by functional groups on the enzyme [4, 5, 6]. By combining substrate binding with catalytic functional groups, small molecules can be transformed into similar catalysts [7, 8, 9, 10]. Traditional artificial enzymes use receptors such as cyclodextrins, crown ethers, and calixarenes to bind substrates [11, 12, 13, 14]. In recent decades, a wide variety of nanomaterials for different applications in sensing [15, 16, 17, 18, 19, 20], catalysis [21, 22, 23, 24, 25, 26, 27, 28, 29, 30, 31], medicine [32, 33, 34, 35, 36], and environmental safety [37, 38, 39, 40, 41, 42, 43] have been synthesized. Among them, there has been a growing interest in nanozymes, which are nanomaterials with intrinsic enzyme‐like properties [44, 45, 46, 47, 48, 49]. These have become popular due to their ability to overcome the limitations of natural enzymes, such as low stability, high cost, and difficult storage [50, 51, 52, 53, 54, 55]. Nanozymes have been extensively studied for various applications, including biosensing [56, 57, 58, 59, 60, 61], COVID‐19 diagnosis [62, 63, 64, 65, 66, 67], and body imaging [68, 69, 70]. With the rapid development and increasing understanding of nanoscience and nanotechnology, nanozymes are expected to act as direct replacements for traditional enzymes by mimicking and engineering the active sites of natural enzymes. In 2007, researchers discovered that Fe_3_O_4_ nanoparticles possess intrinsic peroxidase‐mimicking activity. Since then, hundreds of nanomaterials have been found to mimic the catalytic activity of various enzymes, including peroxidase, oxidase, catalase, haloperoxidase, glutathione peroxidase, uricase, methane monooxygenase, hydrolase, and superoxide dismutase [71, 72, 73, 74, 75, 76].

Among different types of nanozymes, metal–organic frameworks showed many unique characteristics, for instance, abundant catalytic sites, porous crystalline structure, structural tunability, and high surface area, making them attractive candidates for constructing enzyme‐mimicking nanomaterials, commonly referred to as MOFzymes [77, 78, 79]. In fact, MOFzymes integrate the catalytic functionality of enzymes with the robustness and design flexibility of MOF architectures [79]. Hence, the MOFzymes can be used in developing high powerful biosensing platforms, catalytic systems, environmental remediation protocols, and biomedical methods [80]. The catalytic behavior of MOFzymes typically arises from the metal centers or coordinated functional groups within the framework. Transition metal ions such as Fe, Cu, Co, and Mn commonly act as active sites capable of mimicking oxidoreductase enzymes [76, 77, 78, 79, 80]. MOFzymes have shown considerable potential in biosensing applications, where their enzyme‐like activity can be used for signal amplification in colorimetric and electrochemical detection systems [77, 78, 79, 80, 81]. In environmental science, MOFzymes have been explored for pollutant degradation, detoxification of hazardous compounds, and water purification [77, 78, 79, 80, 81]. Despite these advantages, several challenges remain in the development of MOFzymes such as their relatively low catalytic efficiency and substrate selectivity compared with natural enzymes, which restricts their performance in sensitive biosensing and biocatalytic systems. Therefore, developing MOFzyme architectures with improved active‐site exposure, enhanced catalytic efficiency, and better compatibility with multifunctional nanomaterials represents a critical direction for advancing their practical applications.

In this work, a needle‐like MOFzyme with high aspect ratio and extended pH/temperature working ranges was developed for quantification of total antioxidant capacity (TAC) and oxidant‐/light‐free dye degradation via pH‐ and temperature‐independent protocols. The needle‐like MOFzyme was characterized for its size, morphology, surface characteristics, and catalytic activity, and so on. The needle‐like MOFzyme showed a high oxidase‐like activity toward TMB oxidation with an enhanced *V*_max_ and an improved *K*_m_. The stability studies of the needle‐like MOFzyme showed a wide pH range and an extended thermal range. The needle‐like MOFzyme was then utilized for the determination of TAC using trolox as a model antioxidant. Under optimal experimental conditions, the linear range and limit of detection were determined. The selectivity studies were performed, and the inter‐day and intra‐day reproducibility assays were examined to assess the reproducibility of the sensor. The dye degradation was performed using rhodamine B as a model molecule, revealing high degradation efficiency and high cycling stability. The results revealed the applicability of the MOFzyme for real food and environmental applications.

## 2 Experimental Section

### 2.1 Materials

Isopropylamine, MnCl_2_, MeOH, acetic acid, TMB, DAB, OPD, DMSO, Trolox, and rhodamine B dye were obtained from Merck. All metal inorganic salts used for selectivity studies, glucose, ascorbic acid, GSH, cysteine, tryptophan, phenylalanine, aspartic acid, arginine, methionine, caffeine, sucrose, and catechin hydrate were provided from Sigma‐Aldrichin their analytical or synthesis grads and used without any purification.

### 2.2 Synthesis of Mn‐MOF Nanozyme

Briefly, 2.0 mmoL isopropylamine and 1.0 mmoL MnCl_2_ were introduced into 40.0 mL of 50.0%v/v of MeOH aqueous solution, followed by stirring for 15.0 min and then heating at 140°C for 24 h. After that, the oxidase‐like manganese‐based metal–organic frameworks were collected, washed with 50.0%v/v of MeOH, and then dried.

### 2.3 Evaluation of Oxidase‐Like Activity and Kinetic Studies

Oxidase‐like activity was evaluated using the TMB system. In a typical assay, 800.0 μL of acetate buffer (0.10 M, pH 4.0) was mixed with 100.0 μL of TMB solution (10 mM in DMSO), followed by the addition of 50.0 μg mL^−1^ MOFzyme to initiate the reaction. The mixture was incubated at 25°C for 10.0 min, and the absorbance at 652.0 nm was recorded using an ultraviolet–visible (UV–Vis) spectrophotometer. The relative activity was calculated using the following formula [82, 83, 84]

R%=(Activity/Maximum activity)×100

The nonlinear Michaelis–Menten kinetic model was used for the investigation of kinetic factors of the oxidase‐like manganese‐based metal–organic frameworks, that is, *K*_m_ and *V*_max_ [85, 86, 87, 88]. To evaluate the accurate values of the kinetic factors, the linear Lineweaver–Burk model [89, 90, 91, 92] was used.

### 2.4 Sensing Procedure for Total Antioxidant Capacity

The needle‐like MOFzyme was then utilized for the determination of TAC using trolox as a model antioxidant based on a previously reported method [93]. A 50.0 μg mL^−1^ MOFzyme was mixed with 100.0 μL of TMB solution (10.0 mM), and the mixture was incubated for 10.0 min at 25°C. After stopping color growth, 100.0 μL of trolox solution at various concentrations (0.0–200.0 μM) was added to the system, to initiate reduction of the TMB‐ox. The reaction was followed for 10 min at room temperature, the absorbance at 652.0 nm was measured, and the corresponding calibration curve was constructed by plotting the absorbance versus antioxidant concentrations.

Selectivity of the biosensor for TAC detection was evaluated for potential interferents, including biological sugars, metal ions, and amino acids, each at a concentration of 500.0 μM, utilizing the sensing procedure described above.

### 2.5 Dye Degradation Protocol

To do this, 200.0 μg of the MOFzyme was introduced to a 15.0 mg L^−1^ rhodamine dye, and the degradation process was probed by determination of the absorbance of the dye at 555 nm at different time intervals. Notably, the dye solution was used without any pH adjustment, and the degradation process was performed at ambient conditions.

The reusability of the MOFzyme toward the degradation of dye was also assessed. To do this, 0.2 mg MOFzyme was used for the degradation of 15.0 mg L^−1^ dye. After each cycle, the mixture was centrifuged at 15 000 rpm, and the MOFzyme was then collected, washed three times with deionized water, and used for the next cycle.

Regarding the real water samples for dye degradation tests, a river water sample with a pH equal to 7.5, a chlorine concentration of about 1.8 ppm, and a total hardness of about 463 mg L^−1^ per CaCO_3_ was utilized. All samples were directly used with no additional pretreatment to spike different concentrations of dye into the samples, waiting for 24 h, and then the dye was degraded using the developed method.

## 3 Results and Discussion

### 3.1 Characterization of Needle‐Like MOF

#### 3.1.1 Morphological Characteristics

**FIGURE 1:**
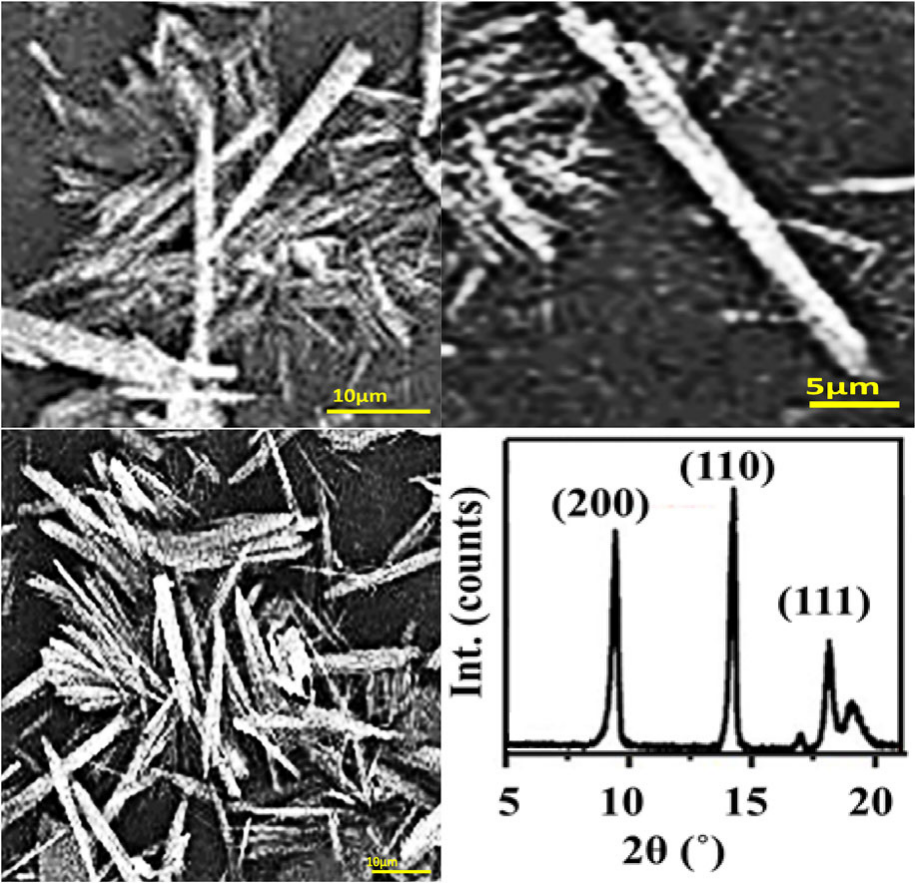
SEM images and XRD spectrum of needle‐like MOFzymes in different magnifications.

The morphology of the synthesized MOFzyme was investigated using scanning electron microscopy (SEM). The SEM images of MOFzyme at different magnifications are shown in Figure 1. The SEM images reveal that the material is composed predominantly of anisotropic microcrystals with well‐defined elongated geometries. The particles exhibit a needle‐like morphology, characterized by high aspect ratios and faceted surfaces, indicating directional crystal growth during the synthesis process. The needle‐like morphology of the MOFzymes plays an important role in their catalytic performance; in fact, the needle‐shaped structures typically exhibit a higher aspect ratio, which can increase the exposure of accessible surface sites and facilitate more efficient interaction between the catalyst and reactant molecules. Therefore, the formation of needle‐like MOF structures is not only a morphological characteristic but also a factor that can influence the overall catalytic behavior of the material.

In addition to isolated crystallites, partial aggregation and bundle‐like assemblies were observed, likely originating from oriented attachment during crystal growth or from capillary forces during solvent evaporation in sample preparation. The pronounced anisotropic morphology suggests preferential growth along a specific crystallographic direction, which is commonly observed in MOFs where strong metal–ligand coordination and ligand geometry promote one‐dimensional crystal extension. Such morphologies are often associated with enhanced mass transport properties and increased exposure of active crystal facets, which may be advantageous for applications in sensing, catalysis, or adsorption.

The X‐ray diffraction (XRD) spectrum of the as‐prepared MOF was also recorded to find the crystalline characteristics of the MOFzyme, and the results are shown in Figure 1; as shown in the figure, the MOFzyme showed sharp and intense diffraction peaks, revealing its high crystallinity. Besides, the characteristic peaks related to the diffraction plans of (200), (110), and (111) of the Mn‐MOF are observable in the XRD pattern, revealing its successful synthesis.

#### 3.1.2 Size Distribution and Aspect Ratio Calculations

Figure S1 shows the size distribution histogram based on the MOFzyme length, showing that the individual MOF crystallites display lengths ranging from approximately 5–40 µm. Based on statistical estimation from SEM observations, the average particle length was determined to be approximately 17 ± 8 µm. Notably, the majority of particles fall within the 10–25 µm length range, suggesting a relatively narrow size distribution centered in the micrometer regime. Figure S2 shows the size distribution histogram based on the MOFzyme width, revealing that their widths are significantly smaller than their length, typically in the range of 0.5–3 µm with an average particle width of 1.5 ± 0.6 µm. The aspect ratio (length‐to‐width ratio, L/W) of the MOFzyme crystallites was evaluated based on SEM observations to assess the degree of anisotropic crystal growth. Figure S3 shows the histogram of the acceptance ratio of the needle‐like MOFzyme. The particles exhibit predominantly high aspect ratios, confirming the elongated morphology observed in the micrographs. The estimated aspect ratio values range from approximately 3 to >15, indicating a broad but clearly anisotropic particle population. As shown in Figure 3a, the majority of the MOFzyme crystallites possess aspect ratios between 5 and 10, accounting for approximately 40% of the total population. In addition, a significant fraction of particles (≈30%) exhibit higher aspect ratios in the range of 10–15, corresponding to well‐defined needle‐like crystallites. Shorter needle‐like particles with aspect ratios between 3 and 5 constitute a smaller portion of the population (≈15%), while highly elongated crystallites with aspect ratios exceeding 15 represent approximately 15% of the observed particles. The predominance of particles with aspect ratios >5 highlights the strong anisotropic growth behavior of the MOFzyme, which is likely governed by preferential coordination and directional extension along a specific crystallographic axis. This anisotropic morphology may therefore play an important role in determining the functional performance of the material in applications such as sensing and catalytic dye degradation.

#### 3.1.3 Nanozymatic Activity of MOFzyme

**FIGURE 2:**
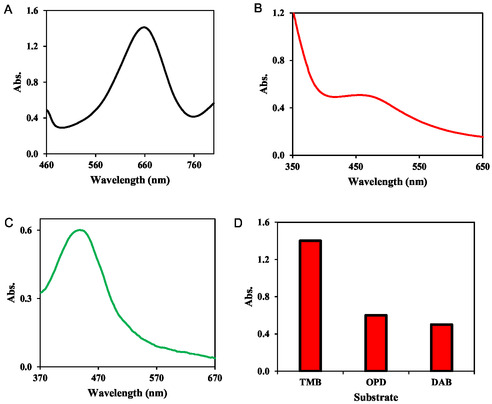
The nanozymatic activity of the MOFzyme toward oxidation of TMB (a), DAB (b), OPD (c), and corresponding absorbance at *λ*_max_ of each substrate (d).

The enzyme‐like (herein, oxidase‐like) behavior of the MOFzyme was investigated by probing its ability for oxidation of the enzyme substrate, TMB (Figure 2A). The as‐prepared MOFzyme can efficiently catalyze the oxidation of TMB in the absence of hydrogen peroxide, revealing its intrinsic oxidase‐like properties. Besides, the versatility of the developed MOFzyme was checked for oxidizing other peroxidase substrates, including DAB and OPD. The results are shown in Figure 2B,C; as can be seen, the MOFzyme can also oxidize both DAB (*λ*_max_ of 460.0 nm) and OPD (*λ*_max_ of 450.0 nm). This investigation proved the versatility of the MOFzyme. Figure 2D shows the absorbances at the *λ*_max_ of each substrate, revealing maximal activity for TMB oxidation; hence, TMB was used for the next experiments.

### 3.2 Kinetic Analysis

**FIGURE 3:**
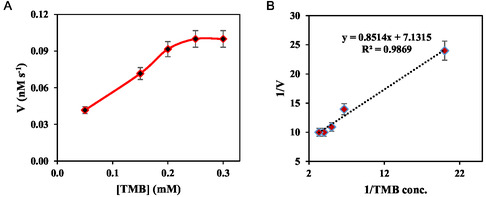
The nonlinear Michaelis–Menten kinetics of the MOFzyme (a) and the linear Lineweaver–Burk kinetics results of the oxidase‐like the MOFzyme (b). All error bars are represented as mean ± standard deviation (*n* = 3).

The kinetic factors of the oxidase‐like manganese‐based metal–organic frameworks, that is, both *K*_m_ (affinity factor) and *V*_max_ (maximal velocity), were estimated toward the oxidation of TMB. To do this, initially, the nonlinear Michaelis–Menten kinetics was applied to describe the kinetic behavior of the oxidase‐like manganese‐based metal–organic frameworks. The Michaelis–Menten saturation curve was constructed by plotting the reaction rate in nM s^−1^ against the TMB concentration in terms of mM. The curve is shown in Figure 3A. Considering the Michaelis–Menten data, the oxidation rate was increased by increasing the TMB concentration, maximized around 0.1 mM, and then leveled off. Statically analysis of Michaelis–Menten data revealed that a V_max_ of 0.111 nM s^−1^ was provided for the oxidase‐like manganese‐based metal–organic frameworks, showing their high catalytic activity. Besides, the *K*_m_ value was found to be around 0.125 mM. In addition, the *V*_max_/*K*_m_ ratio (a crucial indicator of enzyme catalytic efficiency) for the oxidase‐like manganese‐based metal–organic frameworks was estimated to be about 8.9 × 10^−7^ s^−1^ from the nonlinear Michaelis–Menten curve.

The Lineweaver–Burk kinetics model was also employed for accurate and more precise calculation of the kinetic factors of the oxidase‐like manganese‐based metal–organic frameworks. The results are represented in Figure 3B, providing a linear line with a first‐order equation of *y* = 0.8514x + 7.1315 and an *R* ^2^ as high as 0.9869. The high value of *R* ^2^ of the Lineweaver–Burk plot points to the fact that the Lineweaver–Burk kinetics model can successfully describe the kinetic behavior of oxidase‐like manganese‐based metal–organic frameworks. According to Lineweaver–Burk plot, a *V*_max_ of 0.1402 nM s^−1^ was provided for the oxidase‐like manganese‐based metal–organic frameworks, which is in good agreement with that provided from the nonlinear Michaelis–Menten plot. Besides, the *K*_m_ value of about 0.12 mM was also estimated for the nanozymes based on the Lineweaver–Burk plot. The difference between the *K*_m_ values obtained from nonlinear and linear models is as small as 0.005 mM, which can be attributed to common random errors of the calculations. Moreover, the value of the *V*_max_/*K*_m_ ratio from the Lineweaver–Burk plot was estimated as 1.8 × 10^−6^ s^−1^, which is close to the value obtained from the Michaelis–Menten curve.

Notably, the summary of the kinetic parameters of the oxidase‐like manganese‐based metal–organic frameworks provided from both linear Lineweaver–Burk and nonlinear Michaelis–Menten kinetics models is represented in Table S1 to provide a better insight for comparing the results of these models. The kinetic factors of MOFzyme were compared with those of other nanozymes (Table 1). As can be seen, the MOFzyme revealed a comparable *V*_max_ compared to the other nanozymes, revealing its high catalytic efficiency. The MOFzyme revealed a *K*_m_ value exactly lower than the *K*_m_ value of the other nanozymes, revealing the higher substrate affinity.

**TABLE 1:** Comparing the kinetic parameters of MOFzyme with other nanozymes.

Nanozyme	*K*_m_, mM	*V*_max_, M s^−1^	Ref.
NiFe_2_O_4_ nanozymes	8.4	8.6 × 10^−9^	[27]
SiO_2_‐supported gold nanozymes	0.05	2.1 × 10^−8^	[23]
ZnFe_2_O_4_ nanozymes	22.6	8.2 × 10^−9^	[60]
BSA‐gold nanoclusters	25.3	7.2 × 10^−8^	[28]
CuCo‐MOF	0.101	1.2 × 10^−8^	[94]
Multimetallic MOF^a^	0.1	4.7 × 10^−9^	[95]
Hydrogel‐coated Mn‐Zr‐MOFzyme	2.0	9 × 10^−8^	[96]
Developed MOFzyme	0.12	1.4 × 10^−8^	**This work**

a Including five transition metals (Fe, Co, Ni, Cu, and Zn).

### 3.3 Optimizing the Nanozymatic Activity of MOFzyme

#### 3.3.1 pH Working Range

**FIGURE 4:**
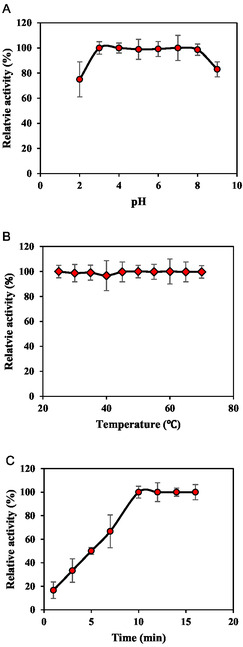
The effect of pH (a), temperature (b), and time (c) on the relative activity of the MOFzyme. All error bars are represented as mean ± standard deviation (*n* = 3).

The effect of pH on the relative activity of the MOFzyme was checked by quantifying their activity in different pH levels varying from 2.0 to 9.0, while the relative activity of the MOFzyme was calculated using the following formula, based on the previously reported literature [19, 20, 21, 22]: Relative activity (%) = (activity/maximum activity) × 100. The results are shown in Figure 4A; the maximum activity of the MOFzyme was found to be over 3.0–8.0. However, at pH = 9, the MOFzyme retained 83% of its activity. As can be seen from the pH effect evaluations, the MOFzyme exhibits stable catalytic activity over a wide pH range, demonstrating its strong environmental tolerance and robustness. This broad pH adaptability is advantageous for practical applications because it allows the nanozyme to operate effectively in different sample conditions, including complex biological and food matrices, without significant loss of activity.

#### 3.3.2 Temperature Working Range

The thermal stability of oxidases, like other proteins, is influenced by various factors and can vary significantly between enzymes. Generally, oxidases tend to lose their activity and denature at higher temperatures. The traditional method uses some strategies, like immobilization or the addition of stabilizing agents to increase their thermal stability. However, the immobilization leads to inactivation or loss of activity of the enzyme compared to the native free enzyme. As an alternative way, the developing nanozymes with oxidase‐like activity can be used for designing highly stable oxidases. Hence, the thermal stability of the oxidase‐like MOFzymes was evaluated as one of the most important factors affecting their practical applications. To do this, the effect of temperature on the relative activity of the oxidase‐like manganese‐based metal–organic frameworks was examined by quantifying their activity at different temperatures over 25–70°C. The results are shown in Figure 4B, revealing that the oxidase‐like manganese‐based metal–organic frameworks can save their maximal activity over a wide thermal range of 25–70°C. It should be noted that these results proved that the developed MOF nanozyme maintains stable catalytic performance over a wide temperature range, indicating excellent thermal stability. This robustness allows the nanozyme to function reliably under varying experimental and environmental conditions, which enhances its suitability for practical applications such as biosensing and antioxidant detection in real samples.

#### 3.3.3 Time‐Dependent Experiments

The enzyme‐like (herein, oxidase‐like) behavior of the MOFzyme under time‐dependent experiments was investigated by calculation of its activity at different time intervals (Figure 4C). By increasing the time, the activity of the MOFzyme was increased, reached its maximal value after 10.0 min, and then leveled off. Hence, 10.0 min was selected as the optimal reaction time for the nanozymatic oxidation of TMB by MOFzyme.

### 3.4 Determination of Total Antioxidant Capacity

#### 3.4.1 Calibration Curves and Limit of Detections

**FIGURE 5:**
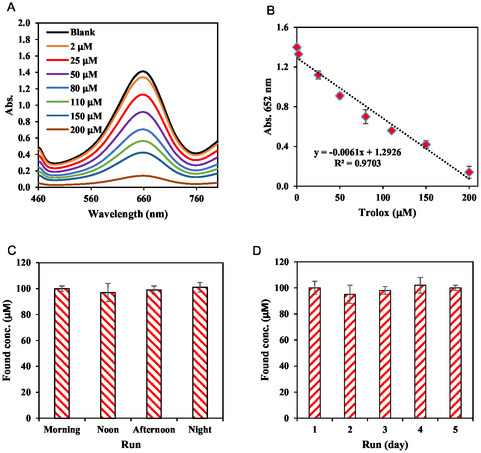
UV–Vis spectra of oxidation product in the presence of different concentrations of antioxidant compound (a) and calibration curve for TAC (b), intra‐day reproducibility (c), and inter‐day reproducibility (d). All error bars are represented as mean ± standard deviation (*n* = 3).

At optimal conditions for maximal activity of the MOFzyme, a pH‐ and temperature‐independent sensor was developed for quantification of TAC. To do this, MOFzyme was incubated with TMB solution, and the mixture was incubated for 10 min at 25°C. After stopping color growth, trolox solution at various concentrations (0–200 μM) was added to the system to initiate reduction of the TMB‐ox. The reaction was followed for 10 min at room temperature, and the absorbance at 652 nm was then measured. Figure 5A shows the UV–Vis spectra of TMB‐ox in the presence of trolox solution at various concentrations (0–200 μM), revealing a decrease in the absorbance at 652 nm by increasing the antioxidant concentration. Besides, the corresponding calibration curve was constructed by plotting the absorbance versus antioxidant concentrations, providing a linear range of 0.0–200 μM for TAC quantification using the developed MOFzyme (Figure 5B). The LOD of the biosensor was calculated utilizing the 3s law, where the LOD is defined as 3s_b_/m (*s*_b_ is the standard deviation of blank and *m* is the slope of the calibration curve). Using the 3s law, the LOD of the biosensor was calculated to be about 1.0 μM, which is significantly lower than the LOD of previously reported methods (Table 2).

**TABLE 2:** Figures of merit of the MOFzyme biosensor compared to the reported methods for TAC quantification.

Method	Model antioxidant	LOD, μM	Sensitivity, L μmol^−1^	LDR, μM	Ref.
ABTS‐based paper‐based sensor	trolox	‐‐‐	0.002	0.0–160.0	[93]
Fe_3_O_4_@Ag@Pt	Ascorbic acid	5.09	0.003	0.0–300.0	[97]
Fe_3_O_4_@Ag@Pt	Glutathione	1.97	0.009	0.0–200.0	[97]
Fe_3_O_4_@Ag@Pt	trolox	8.99	0.0019	0.0–500	[97]
MOFzyme biosensor	trolox	1.0	0.006	0.0–200.0	**This work**

#### 3.4.2 Reproducibility

The reproducibility of the sensor was checked by both intra‐day and inter‐day assays [61, 98]. In all tests, the antioxidant concentration was fixed to 100.0 μM. The results are shown in Figure 5C, D. As can be seen from the Figure 5C, the intra‐day reproducibility was checked in different times within a day, including morning, noon, afternoon, and night to prove the reproducibility of the results in different time intervals of a day, and the results revealed an extremely low RSD% of 1.7% for the developed pH‐ and temperature‐independent sensor. The low RSD% of the sensor showed the accuracy of the developed MOFzyme biosensor.

Besides the intra‐day reproducibility, the inter‐day reproducibility of the sensor was also checked to prove the accuracy and reliability of the developed sensor. It is well known that reliable sensors should provide approximately the same signals on different days to prove their accuracy and reliability. The main approach for evaluating this is inter‐day reproducibility. Hence, in this regard, the inter‐day reproducibility of the developed MOFzyme biosensor was investigated within 5 days by determining a constant antioxidant concentration of 100.0 μM. The results showed that a very low inter‐day RSD% of about 2.39% was provided for the biosensor, revealing its high reproducibility (Figure 5D). Regarding the reliability and accuracy of the results, the mean concentration found in 5 days was calculated to be about 99.0 ± 2.36 μM, exhibiting that the estimated concentration is exactly close to the true value (100.0 μM). Hence, it can be deduced that the intra‐day and inter‐day reproducibility assays showed high reproducibility and reliability of the results.

#### 3.4.3 Selectivity

**FIGURE 6:**
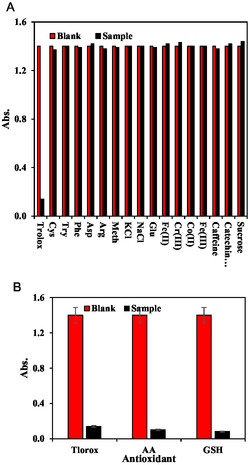
Selectivity of the MOFzyme biosensor (a) and biosensor response toward different antioxidant compounds (b). All error bars are represented as mean ± standard deviation (*n* = 3).

The selectivity of the biosensor was evaluated by checking the sensor response in the presence of different materials, for instance, several metal ions, sugars, and amino acids. The results showed the signal remained constant in the presence of the different sugars and metal ions, revealing high selectivity of the developed MOFzyme biosensor toward quantification of the TAC. Regarding the amino acids, several amino acids as the main similar materials with the antioxidants were checked. The concentration of all amino acids was fixed at 500.0 μM, while the trolox was 50 μM, showing that amino acids cannot result in a significant change in the biosensor analytical signal, demonstrating the high selectivity of the sensor (Figure 6A). Besides, the most common potential interferents commonly found in tea including caffeine, sucrose, and catechin hydrate were also checked at a fixed concentration of 500.0 μM, showing no interference (Figure 6A). According to the results of the selectivity assessments, the developed MOFzyme demonstrates high selectivity, showing negligible interference from common coexisting species such as amino acids, sugars, inorganic ions, caffeine, and sucrose. This excellent selectivity ensures reliable detection of target antioxidants even in complex matrices. Furthermore, the successful application of the system to tea as a real sample for TAC detection confirms its practical applicability and analytical reliability in real‐world food and beverage analysis.

#### 3.4.4 Sensor Response in the Presence of Different Antioxidants

Although in this work, trolox was utilized as a model antioxidant for determining the figures of merit of the MOFzyme biosensor, in real media, there are several different antioxidants; for instance, two of the most important antioxidants are ascorbic acid and glutathione. Hence, the sensor response toward the ascorbic acid and glutathione was also evaluated at a fixed concentration of 50.0 μM. The results of this experiment are shown in Figure 6B, revealing high sensitivity of the MOFzyme biosensor toward different antioxidants, showcasing that the MOFzyme is suitable for sensitive and reliable quantification of TAC. The strong catalytic response of the developed MOFzyme toward the antioxidants ascorbic acid (AA) and glutathione (GSH) indicates its high sensitivity to biologically relevant reducing agents. This capability is important because both AA and GSH play critical roles in cellular redox balance and oxidative stress regulation. Notably, the glutathione signaling was also confirmed by Hormozi‐Jangi multinanozyme glutathione assay (HMGA) [47] as the reference method, confirming our results. Therefore, the nanozyme shows potential for applications in antioxidant detection, biosensing, and biomedical analysis related to oxidative stress and food quality control.

#### 3.4.5 Real Sample Analysis

The TAC of different types of tea was determined by the developed MOFzyme biosensor using the standard addition method. The results are shown in Table 3, revealing that the biosensor shows a very good ability toward the determination of the TAC in food samples. To provide a more precise insight into the ability of the sensor for quantification of the TAC of tea samples, the results are compared with the results provided by the standard kit, and the results in Table 3 are showcasing that the concentrations provided by the developed MOFzyme biosensor are in a good agreement with the results of the standard kit, revealing the high reliability and accuracy of the results provided by the developed MOFzyme biosensor, recovery of 95.2–104.3% along with a very low RSD% of 2.7–3.6%.

**TABLE 3:** Real sample analysis for the TAC MOFzyme biosensor.

Sample	Found TAC, mmol trolox/L	RSD, %	Found by the standard method, mmol trolox/L	RSD, %	Recovery, %
Green tea	4.0	2.7	4.2	2.2	95.2
Black tea	10.9	3.1	11.3	2.5	96.3
Ice tea	2.4	3.6	2.3	3.5	104.3

### 3.5 Dye Degradation

#### 3.5.1 Dye Degradation Performances

**FIGURE 7:**
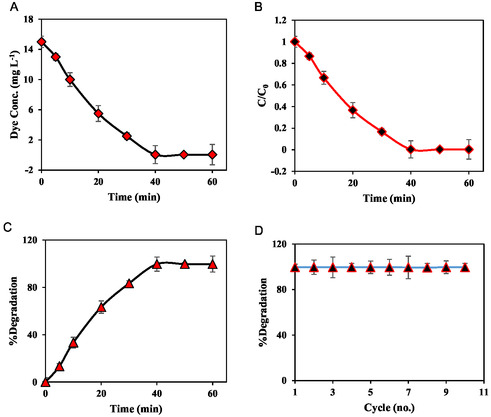
The plot of the dye concentration as a function of degradation time (a), the plot of the C/C_0_ as a function of degradation time (b), the plot of the degradation efficiency as a function of degradation time (c), and the reusability of MOFzyme toward dye degradation (d). All error bars are represented as mean ± standard deviation (*n* = 3).

The MOFzyme synthesized in this work reveals extended pH and thermal ranges, resulting in its applicability for developing pH‐ and temperature‐independent dye degradation protocols; hence, the main affecting factor on the dye degradation efficiency, that is, time, was evaluated on the degradation of dye. The results are shown in Figure 7A–C, revealing that the dye concentration was decreased by increasing the time of incubation with the developed MOFzyme, reached its minimum value after 40.0 min, and after that, increasing the time has no effect on the degradation process (Figure 7A,B). To provide more precise results on the dye degradation efficiency, the plot of the degradation efficiency as a function of degradation time was constructed (Figure 7C), demonstrating that the yield of degradation increased by increasing the time and after 40.0 min, 99.7% of the dye was degraded over the oxidase‐like MOFzyme without using any oxidant or light irradiation, revealing its high ability for sustainable dye degradation. Besides, the kinetics of dye degradation using the developed method was checked by the first‐order kinetics model (Figure S4), revealing a *K*_app_ of 0.0658 min^−1^ (*R* ^2^ of 0.9928) for dye degradation over the developed oxidase‐like MOFzyme.

#### 3.5.2 Reusability

Reusability of a nanozyme or MOFzyme is one of the most significant features of these materials toward real‐world applications [22, 68, 69, 70, 71]; hence, the reusability of the MOFzyme toward the degradation of dye was assessed. To do this, after each cycle, the MOFzyme was collected via centrifuge, washed with DI water, and used for successive experiments (Figure 7D), exhibiting that the MOFzyme retained their maximal activity for at least 10 cycles. Demonstrating that the MOFzyme retains its catalytic activity for 10 consecutive cycles highlights its excellent operational stability and reusability. This recyclability is important because it reduces material consumption, lowers experimental and operational costs, and improves the practicality of the nanozyme for real applications. In addition, stable performance over multiple cycles indicates that the nanostructure maintains its structural integrity and active sites without significant deactivation or leaching, which is critical for long‐term catalytic and sensing applications.

#### 3.5.3 Dye Degradation From Real Samples

The applicability of the developed method for the dye degradation from the real media was assessed by degrading different concentrations of rhodamine B over 5–25 mg L^−1^ from river water. The results are shown in Table 4, revealing a degradation efficiency over 98.1–99.8% along with a very low RSD% of 2.9–5.6%. Besides, a textile waste water containing 200 mg L^−1^ rhodamine B was also exposed to the developed MOFzyme to degrade the rhodamine B, revealing a degradation efficiency of 97.5%. The results of this investigation revealed that the MOFzyme developed in this study is suitable for sustainable dye degradation from real media without needing any oxidant or light irradiation.

**TABLE 4:** Dye degradation using the developed MOFzyme from river water.

Sample	Dye concentration, mg L^−1^	Degradation, %	RSD, %
River water	5	99.8	3.7
	10	99.5	5.6
	15	99.7	4.3
	20	98.7	2.9
	25	98.1	3.5

### 3.6 Proposed Mechanism

The mechanism of behind the oxidase‐like activity of the as‐prepared MOF nanozyme was assessed by radical scavenging experiments. In this regard, the dye degradation was performed in the presence and the absence of different radical scavengers including benzoquinone and t‐BuOH as the superoxide and hydroxyl‐radical scavengers (Figure S5), and the results revealed that the reaction was significantly inhibited by benzoquinone, while the effect of t‐BuOH was found to be minimal on the catalytic process. Hence, the superoxide species that resulted from the oxygen activation over the MOFzyme are the main contributors in the catalytic reaction. To confirm the fact that the oxidase‐mimetic activity of MOFzyme derived from O_2_ activation, the UV–Vis spectra of the Mn MOF + TMB system under N_2_ and air atmospheres were recorded (Figure S6), showing significant inhibition of the catalysis under N_2_ atmosphere. Hence, one can deduce that the oxidase‐mimetic activity of MOFzyme is derived from O_2_ activation. In fact, the O_2_ was converted to the active superoxide species over the Mn‐MOFzyme and then the superoxide reacted with the dye molecules, resulted to their decolonization. Notably, regarding the TMB oxidation, the mechanism is same. In this case, the produced superoxide species was reacted with the TMB molecules and the blue‐colored TMB radical cation will be produced as the oxidation product which can be used as the analytical probe [29, 30, 41].

## 4 Conclusions

A needle‐like MOFzyme was synthesized and characterized, revealing a high aspect ratio and extended pH and temperature working ranges (pH range of 3.0–8.0 and an extended thermal range of 25–70°C). The MOFzyme showed high oxidase‐like activity with an enhanced *V*_max_ of 0.14 nM s^−1^ and an improved *K*_m_ of 0.12 mM, making it suitable for developing a biosensor toward quantification of TAC. Under optimal experimental conditions, a linear range of 0.0–200.0 μM, a very low detection limit of 1.0 μM, and high selectivity against coexisting materials were obtained for the biosensor. Besides, the MOFzyme developed in this study was found to be suitable for sustainable dye degradation from real media without needing any oxidant or light irradiation. The dye degradation was performed using rhodamine B as a model molecule, revealing 99.7% degradation within 40.0 min without any oxidant or light irradiation, along with at least 10 cycles without reduction of its activity. As a consequence, this method can be utilized for real‐world applications toward the food safety control and sustainable treatment of drinking water resources.

## Supporting Information

Additional supporting information can be found online in the Supporting Information section.

## Conflicts of Interest

The authors declare no conflicts of interest.

## Supporting information

Supplementary Material
