# Supplementary material for: A Needle‐Like MOFzyme with High Aspect Ratio and Extended pH/Temperature Working Ranges for Total Antioxidant Capacity Determination and Oxidant‐/Light‐Free Dye Degradation
Source: ChemistryOpen. 2026 Apr 24;15(5):e70196. doi: 10.1002/open.70196 (PMC13109643; doi:10.1002/open.70196)
Supplement: Supplementary file 1 — Supplementary Material [file OPEN-15-e70196-s001.pdf]

Supporting information

A needle-like MOFzyme with high aspect ratio and extended pH-/temperature-working ranges for antioxidant capacity determination and oxidant-/light-free dye degradation

Hanieh Borhanipasvisheh*[,1] Anahita Barghi[2], Peyman Abazari[3]

[1]*Department of Inorganic Chemistry, Faculty of Chemistry, University of Guilan, P.O. Box 41335–1914, Rasht, Iran; ORCID: 0009-0005-0891-5306 (HB)*

[2]*Institute of Agricultural Life Science, Dong-A University, Busan, 49315, South Korea*

[3]*Department of Analytical Chemistry, Faculty of Chemistry, University of Kashan, Kashan, Iran*

* ***Email:****haniehborhanipasisheh@gmail.com*

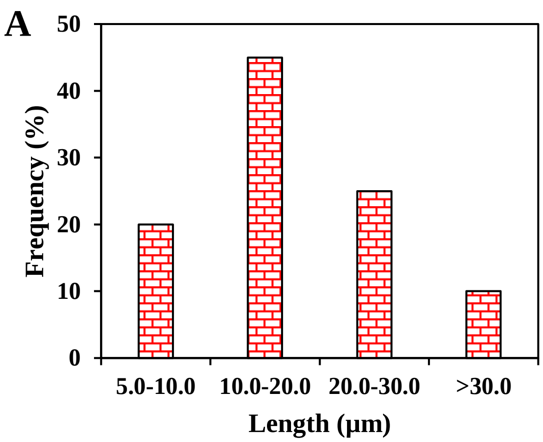

Figure S1. The size distribution histogram based on the MOFzyme length.

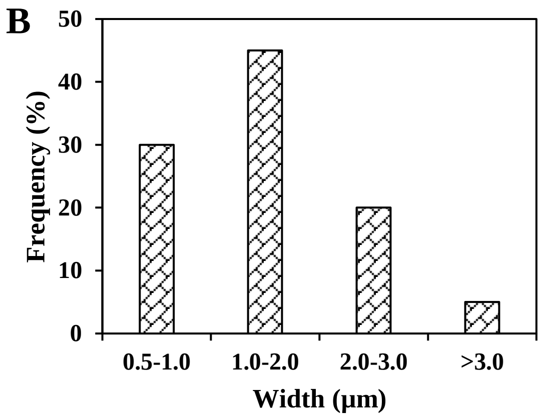

Figure S2. The size distribution histogram based on the MOFzyme width.

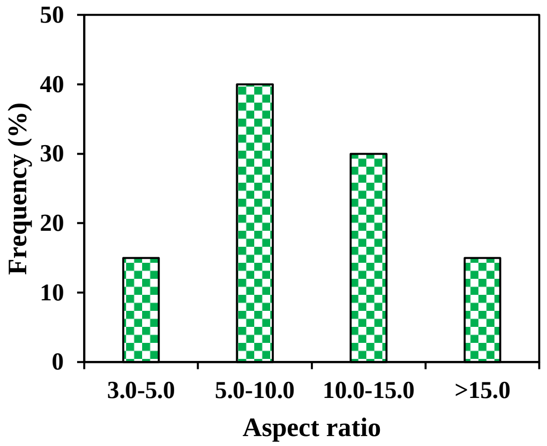

Figure S3. The histogram of the accept ratio of the needle-like MOFzyme.

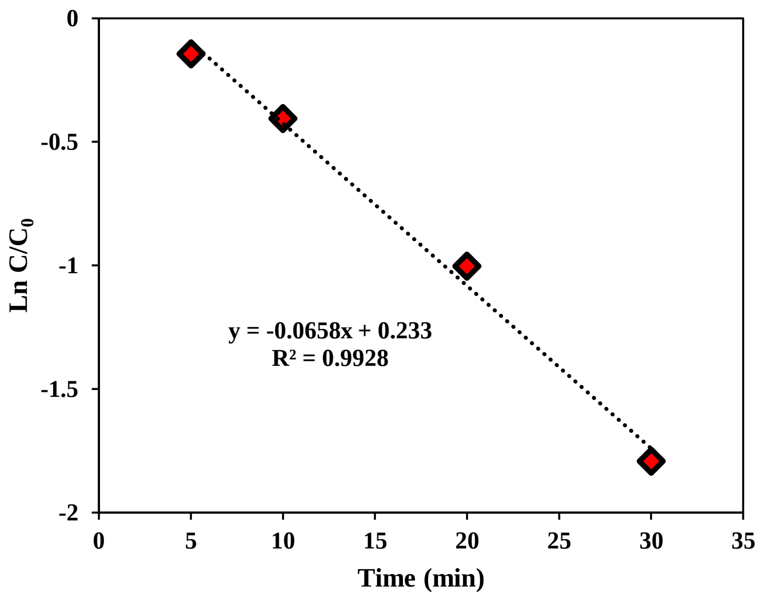

Figure S4. The first-order kinetics of dye degradation using the developed method.

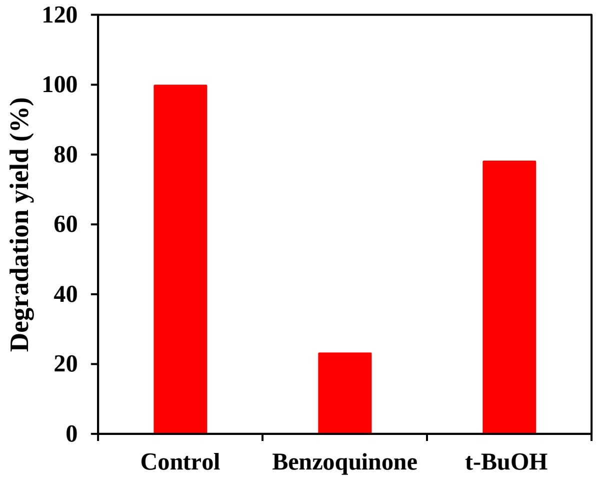

Figure S5. Effect of scavengers on the nanozymatic degradation of rhodamine B over the developed MOFzyme.

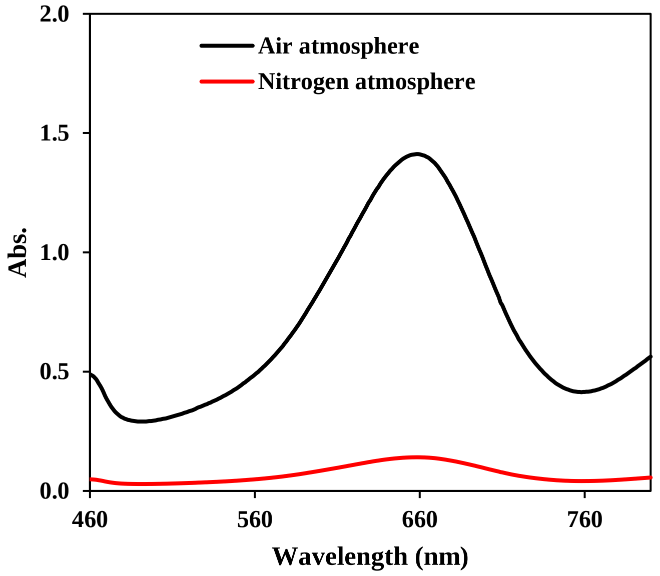

Figure S6. UV-vis spectra of the Mn MOF + TMB system under N_2 and air atmosphere confirmed the oxidase-mimetic activity derived from O_2 activation.

Table S1. The summary of the kinetic parameters of the MOFzyme

Parameter	Unit	Non-linear Michaelis-Menten model	Linear Lineweaver–Burk model
V_{max}	nM s^{-1}	0.111	0.1402
K_m	mM	0.125	0.12
V_{max}/K_m	s^{-1}	8.9×10^{-7}	1.8×10^{-6}
